# A Higher Risk of Acute Rejection of Human Kidney Allografts Can Be Predicted from the Level of CD45RC Expressed by the Recipients’ CD8 T Cells

**DOI:** 10.1371/journal.pone.0069791

**Published:** 2013-07-24

**Authors:** Laurence Ordonez, Isabelle Bernard, Marianne Chabod, Jean-François Augusto, Valerie Lauwers-Cances, Christelle Cristini, Maria-Cristina Cuturi, Jean-François Subra, Abdelhadi Saoudi

**Affiliations:** 1 Inserm, U1043, Toulouse, France; 2 CNRS, U5282, Toulouse, France; 3 Université de Toulouse, Centre de Physiopathologie de Toulouse Purpan, Toulouse, France; 4 Inserm U892, Service de Nephrologie-Dialyse Transplantation CHU Angers and Université d’Angers, Angers, France; 5 The Epidemiology Unit, CHU Toulouse, Toulouse, France; 6 Inserm, U1046, Center of research in Transplantation and Immunology, Nantes, France; University of Oslo, Norway

## Abstract

Although transplantation is the common treatment for end-stage renal failure, allograft rejection and marked morbidity from the use of immunosuppressive drugs remain important limitations. A major challenge in the field is to identify easy, reliable and noninvasive biomarkers allowing the prediction of deleterious alloreactive immune responses and the tailoring of immunosuppressive therapy in individuals according to the rejection risk. In this study, we first established that the expression of the RC isoform of the CD45 molecule (CD45RC) on CD4 and CD8 T cells from healthy individuals identifies functionally distinct alloreactive T cell subsets that behave differently in terms of proliferation and cytokine secretion. We then investigated whether the frequency of the recipients CD45RC T cell subsets before transplantation would predict acute graft rejection in a cohort of 89 patients who had undergone their first kidney transplantation. We showed that patients exhibiting more than 54.7% of CD8 CD45RC^high^ T cells before transplantation had a 6 fold increased risk of acute kidney graft rejection. In contrast, the proportions of CD4 CD45RC T cells were not predictive. Thus, a higher risk of acute rejection of human kidney allografts can be predicted from the level of CD45RC expressed by the recipients’ CD8 T cells.

## Introduction

Transplantation has become a standard medical practice for end-stage organ failure. Nevertheless, allograft rejection represents a common complication, affecting the long-term outcome of the transplanted organ. Many immune cells participate in acute allograft rejection but alloreactive CD4^+^ and/or CD8^+^ T lymphocytes usually play the major role [1,2]. The introduction of immunosuppressive drugs has revolutionized the field of transplantation by substantially reducing the frequency of acute rejection [3,4], but these benefits are dampened by the drugs own toxicity, and by their side effects which include opportunistic infections and virus-induced cancers that have been found to occur at an increased frequency after organ transplantation [5]. The marked morbidity resulting from the long-term use of immunosuppressive drugs remains an important drawback, and it is thus clinically beneficial to limit the amounts of drugs used to the minimum required to control the alloreactive responses leading to organ rejection. Today, a major challenge in the field of transplantation is the identification of easy, reliable and noninvasive markers that would predict the probability of organ rejection. This would help to improve the care of organ allograft recipients and allow individual tailoring of the doses of potentially toxic immunosuppressive drugs being used.

CD45 is a transmembrane protein tyrosine phosphatase that operates as a regulator of kinases belonging to the Src-family kinases and is essential for efficient signal transduction after T cell receptor engagement [6–8]. Several CD45 isoforms differing in size and charge are generated by alternative splicing of exons 4(A), 5(B) and 6(C), leading to changes in the extracellular domain of the molecule [9,10]. The level of CD45 isoforms expression by T cell is highly variable between individuals [11–13] and is genetically predetermined [12–14]. Although CD45 alternative splicing is highly regulated and conserved among vertebrates, the function of the different CD45 isoforms is not clear. However, differential expression of the CD45 isoforms has been associated with different stages of T cell development and function. Recently, it has been shown that subset of human T cells expressing CD45RC exhibit different cytokine profiles after polyclonal stimulation, and that the frequency of these cells is imbalanced in patients with vasculitis [11]. Several groups have shown that, in rodent models, T cells expressing high levels of a particular CD45 isoform (CD45RC in rats or CD45RB in mice) are potent effector cells capable of promoting transplant rejection and organ inflammation [15–18]. In contrast, T cells expressing low levels of that isoform exert a regulatory activity and inhibit allograft rejection [19–21] and autoimmune diseases [15–17,22,23]. In addition, it has been shown that treatment of mice with anti-CD45RB antibodies reliably induced donor-specific tolerance [24,25]. Although these experimental findings have clearly demonstrated that the genetically determined expression of CD45 isoforms on T cells may modulate their rejection potential, the alloreactive properties of these T cell subsets in humans are still unknown.

In the present study, we showed that CD4 and CD8 T cells from healthy humans, separated according to the levels of CD45RC, exhibited different responses to allogeneic stimulation, in terms of proliferation and cytokines secretion. We then investigated whether the frequency of CD45RC T cell subsets in patients before transplantation can help to predict the outcome of kidney transplantation. We found that a higher risk of acute rejection of human kidney allografts can be predicted from the pre-determined level of CD45RC expressed by the recipients’ CD8 T cells.

## Materials and Methods

### Patients and sample collection

#### Patients and healthy individuals

For this prospective study, we selected a cohort of 89 patients who received a first kidney transplant obtained from deceased donors at the University Hospital Center of Angers, France. All patients gave their written consent. Patients that had received multiple organ transplantations or were found to have panel reactive antibodies ≥ 20% were excluded from the study. Peripheral blood mononuclear cells (PBMC) were harvested from peripheral blood samples before transplantation and were stored in liquid nitrogen. Initial experiments showed that the proportion of CD45RC T cell subsets is not affected by freezing/thawing samples in liquid nitrogen (data not shown). For the healthy individuals, peripheral blood mononuclear leucocytes were obtained from Buffy coat preparations drawn from anonymous blood donors, at the Purpan University Hospital blood bank (Toulouse, France). This study was approved by the Medical Ethics Committee of the University Hospital Center of Angers (2009/10) and of the Purpan University Hospital blood bank (EFS-PM n° 21/PVNT/TOU/INSO1/2009-0006).

#### Immunosuppressive regimen

For the recipients of organ transplants, the induction treatment consisted in methylprednisolone 500 mg intravenously on day 0 (Solu-Medrol®, Pfizer, France) alone (n=8), or in association with 20 mg basiliximab intravenously on days 0 and 4 (Simulect®, Novartis, Basel, Switzerland; n=26) or in association with 5-7 day course of antithymocyte globulins (1.5 mg/kg/d, Thymoglobuline®, Genzyme, Lyon, France; n=55). Prednisolone 1 mg/kg/d was given between day 1 and day 14 then 0.5 mg/kg/d followed by a progressive decrease and complete discontinuation at the end of the 5^th^ month. Corticosteroids were not withdrawn for patients with more than one episode of acute cellular rejection or with a previous episode of acute steroid-resistant rejection. In addition, all patients received mycophenolate mofetil (Cellcept®, Roche, France) 2g/day from day 0 and subsequently adapted according to clinical events, and tacrolimus (Prograf®, Fujisawa, Japan). The target level of tacrolimus during the first six months was 8-12 ng/mL and 6-8 ng/mL after. Fifty-seven transplant recipients had tacrolimus monotherapy from the 6^th^ month post-transplantation. Mycophenolate mofetil was continued in the other patients.

#### Prophylactic treatments

Prophylaxis against *Pneumocystis carinii* was administered for three months to all patients using 400mg sulfamethoxazole / 80mg trimethoprim (Bactrim®). All cytomegalovirus (CMV)-negative patients who had received a kidney from a CMV-positive donor received prophylaxis against CMV infection using valacyclovir (Zelitrex®) for 16 weeks (6-8 g/day according to renal function).

#### Diagnosis of acute rejection

Diagnosis of rejection episodes was based on conventional clinical and laboratory criteria (i.e. transient 20% elevation of creatinine level and/or proteinuria, with normal ultrasound examination) and on data obtained after graft biopsy (scored according to the 2005 Banff classification). The 1, 2 and 4-year incidence of rejection in this cohort were respectively 10.1%, 11.2% and 14.6%. The acute episodes were Banff borderline in one case, grade I in five cases, grade II in three cases and grade III in one case. The remaining four cases were clinically diagnosed and successfully treated.

### Antibodies

FITC-, PE-, PE-Cyan5, PE-Cyan7, Alexa 700, Pacific Blue, APC or biotin-conjugated anti-CD4 (RPA-T4), anti-CD8 (RPA-T8), anti-TCRαβ (BW242/412), anti-CD25 (4E3), anti-CD69 (FN 50), anti-CD45RA (HI100), anti-CD45RC (MT2), anti-CD45R0 (UCHL1), anti-CCR7 (3D12), mAbs as well APC-streptavidin and biotinylated MARG-2a were purchased from BD Biosciences, R&D Systems, IQ Product, Miltenyi, Beckman Coulter or eBioscience.

### Flow cytometry analysis

10^6^ cells were incubated with the indicated Abs. Data were collected either on a FACS-Calibur (BD Biosciences) cytometer using the CELLQuest^TM^ software (BD Biosciences) for analysis, or on a LSR-II (BD Biosciences) cytometer using the DIVA software (BD Biosciences) for analysis.

### Purification of CD45RC T cell subsets

Human PBMCs were prepared by gradient centrifugation (MLS-Ficoll, Eurobio) of buffy coats from healthy individuals. CD4 and CD8 T cells were purified by negative selection using CD4 or CD8 negative isolation kits (Dynal). The purity obtained was 95-98% CD4 or CD8 T cells. CD4 T cells were then stained with limiting amounts of FITC-conjugated anti-CD45RC mAb and separated into CD45RC^high^ and CD45RC^low^ cells by positive selection after addition of anti-FITC MACS microbeads (Miltenyi). The resulting purity was always more than 92% for CD45RC^high^ and CD45RC^low^ CD4 T cells. CD8 CD45RC T cell subsets were purified by cell sorting after labeling purified CD8 T cells with anti-CD8 and anti-CD45RC mAbs and separated on a Coulter cell sorter (Epics Altra; Beckman-Coulter). The purity of sorted CD45RC^high^, CD45RC^int^ or CD45RC^low^ CD8 T cell subsets was higher than 97%.

### T cell stimulation, analysis of T cell proliferation and cytokine production

For mixed lymphocyte reactions (MLR), 10^5^ highly purified CD45RC T cell subsets were stimulated with 2.10^5^ mixed irradiated allogeneic APC (T cell depleted PBMC) prepared from 4 healthy donors. For the CD8 T cell subsets, 50 U/ml of recombinant human IL-2 was added to the culture (AbCys). Proliferation was measured by ^3^H-thymidine uptake during the last 18 h of a 4 or 5 days culture period. Supernatants were removed and stored at -20°C for cytokine determination using BD™ Cytometric Bead Array Human Th1/Th2 cytokine kit (BD Biosciences) and ELISA for IL-17 (eBioscience).

### Statistical analysis

The Wilcoxon matched-pairs test was used for comparison of proliferation and cytokine production within CD4 CD45RC T cell subsets and within CD8 CD45RC T cell subsets. For comparisons between controls and recipients, mean comparisons were performed using Student’s t test or Mann-Whitney, depending on the normality of the distribution and comparisons of percentages using the χ^2^ tests or Fisher’s exact test as appropriate. The coefficient of determination R^2^ was used to test if age could explain the variability for the different CD45RC subsets. The discriminant capacity of different subsets of peripheral T cells was investigated using non-parametric estimation of the area under the receiver operating characteristic (ROC) curves (AUC). The different AUC were compared using a Wilcoxon non-parametric test. The different subsets were distinguished using the threshold obtained by the ROC curve or using the median of the distribution. For survival analysis, cumulative survival rates were estimated using the Kaplan-Meier method. The relationship between risk of acute transplant rejection and T cell subset parameters was examined using log rank tests and a Cox proportional hazard analysis model, in which the time scale was the time of study with a time origin defined as the transplant date. A multivariate model was built to take into account potential confounding factors. Proportionality in Cox analysis was tested using Schoenfeld residuals and log-log plot. A p value lower than 0.05 was considered significant. Data analysis was performed using Stata software (*Stata Statistical Software: Release 9*. College Station, TX: StataCorp LP).

## Results

### Human CD4 and CD8 CD45RC T cell subsets present distinct alloreactive properties in vitro

**Figure 1:**
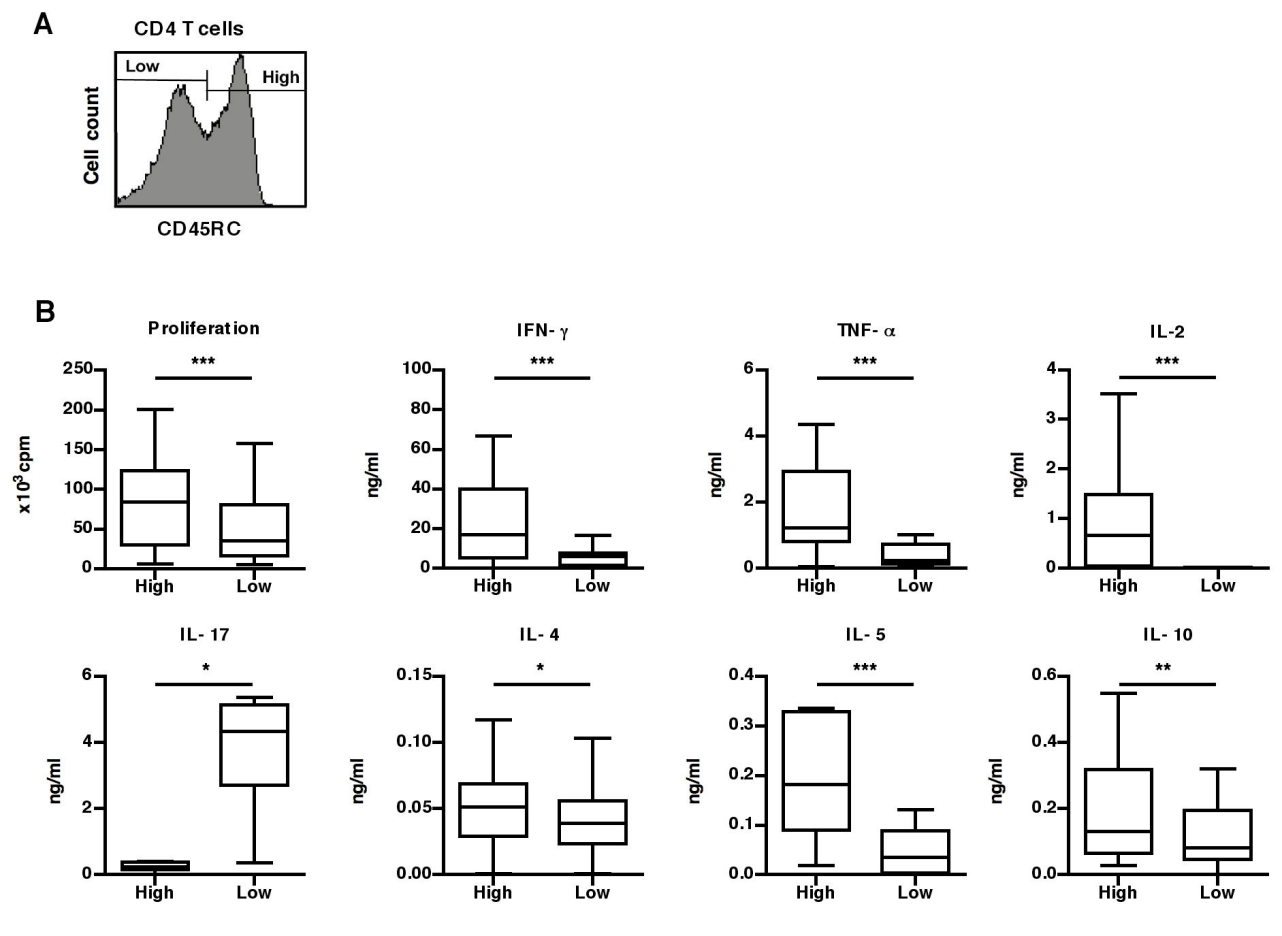
CD45RC expression identifies distinct human CD4 T cell subsets with different alloreactive properties *in vitro*. (A) CD45RC expression on CD4 T cells identifies two CD45RC subsets. (B) Purified CD45RC^high^ (High) and CD45RC^low^ (Low) CD4 T cells were stimulated *in vitro* using T-depleted irradiated allogeneic PBMC from 4 different donors. The proliferation was measured by thymidine incorporation during the last 18h of the 4 days of culture. The supernatants were collected at 96h of culture and analyzed for the presence of cytokines using the BD™ Cytometric Bead Array. The results from 24 healthy individuals are presented as box-plot diagrams. Statistical results comparing the CD45RC^high^ and CD45RC^low^ subsets: proliferation (p=0.0004), IFN-γ (p=0.0005), TNF-α (p=0.0002), IL-2 (p<0.0001), IL-17 (p=0.004), IL-4 (p=0.022), IL-5 (p=0.0017) and IL-10 (p=0.016).

The functional properties of human CD45RC T cells subsets were previously tested *in vitro* using polyclonal activation but their alloreactive potential was still unknown. Flow cytometry analysis of CD45RC expression on CD4 T cells from healthy individuals show a bimodal expression of this membrane marker, allowing the identification of CD45RC^high^ and CD45RC^low^ subsets (Figure 1A). In order to investigate their alloreactive potential, these two sub-populations were purified from peripheral blood of 24 different healthy individuals and stimulated *in vitro* with irradiated allogeneic APC. Upon these conditions of stimulation, CD45RC^high^ CD4 T cells proliferated twice more than CD45RC^low^ CD4 T cells (Figure 1B). Regarding their capacity to secrete cytokines, the CD45RC^high^ subset produced higher amounts of IFN-γ, TNF-α, IL-2, IL-5, IL-4 and IL-10 (Figure 1B), whilst CD45RC^low^ CD4 T cells produced higher amount of IL-17 (median=4.3 vs 0.3 ng/ml). These data demonstrate that two subsets of human CD4 T cells can be distinguished according to their CD45RC expression: the CD45RC^high^ CD4 T cell subset proliferates vigorously to allo-stimulation and produces the majority of the cytokines tested except IL-17, which is mainly produced by the CD45RC^low^ subset (Figure 1B).

**Figure 2:**
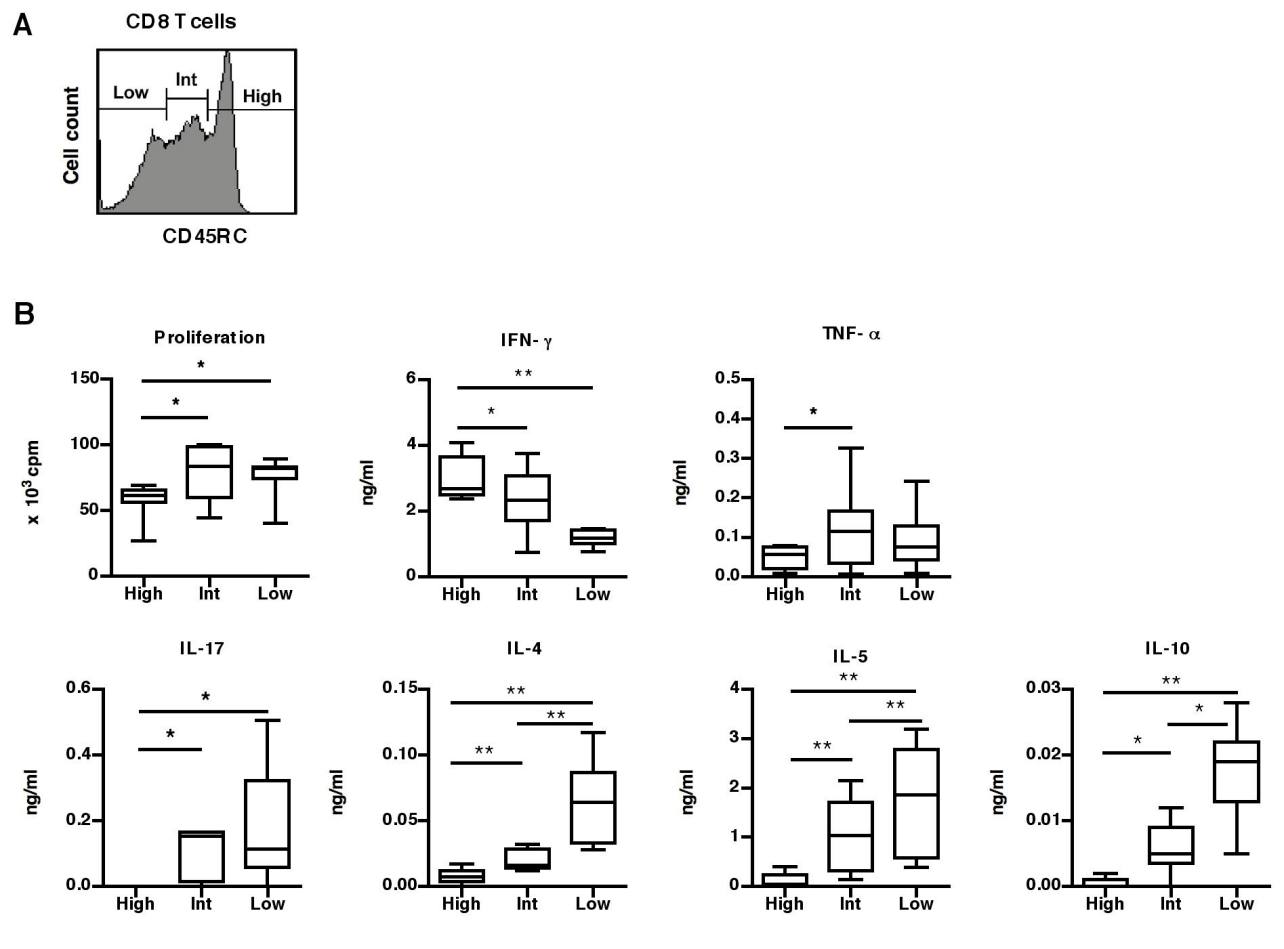
CD45RC expression identifies distinct human CD8 T cell subsets with different alloreactive properties *in vitro*. (**A**) CD45RC expression on CD8 T cells identifies three CD45RC subsets. (**B**) Purified CD45RC^high^ (High), CD45RC^int^ (Int) and CD45RC^low^ (Low) CD8 T cells were stimulated by MLR as described above for CD4 T cells and the proliferation and cytokine analysis were performed at day 5. The results obtained in 7 healthy individuals are presented as box-plot diagrams. Statistical results comparing the CD45RC^high^, CD45RC^int^ and CD45RC^low^ subsets: proliferation (p{high/low}=0.043; p{high/int}=0.043), IFN-γ (p{high/low}=0.018; p{high/int}=0.043), TNF-α p{high/int} = 0.028), IL-17 (p{high/low} = 0.028; p{high/int} = 0.042), IL-4 (p{high/low} = 0.018; p{high/int} = 0.018; p{int/low} = 0.018), IL-5 (p{high/low} = 0.018; p{high/int} = 0.018; p{int/low} = 0.018) and IL-10 (p{high/low} = 0.018; p{high/int} = 0.027; p{int/low} = 0.028). The p-values were calculated using the Wilcoxon matched-pairs test; *, p<0.05; **, p<0.02; ***, p<0.002.

For CD8 T cells, analysis of CD45RC expression allows the definition of three separate subsets: CD45RC^high^, CD45RC^int^ and CD45RC^low^ (Figure 2A). To study their alloreactive properties, these three sub-populations were purified from the peripheral blood of 7 healthy individuals and stimulated using mixed-lymphocyte cultures. Although CD8 CD45RC^high^ T cells proliferated slightly less than the other two subsets, they produced higher amounts of IFN-γ (Figure 2B). Conversely, the CD45RC^low^ subset produced higher amounts of IL-17, IL-4, IL-5 and IL-10. The CD45RC^int^ subpopulation had an intermediate production of IFN-γ, IL-17, IL-4, IL-5 and IL-10. Thus, the level of CD45RC expression divides human CD8 T cells into three subsets with differential alloreactive properties; the alloreactive CD8 T cells responsible for production of IL-17, type 2 and regulatory cytokines are mainly contained within the CD45RC^low^ and CD45RC^int^ subsets.

### The proportion of CD45RC subsets is highly variable between individuals

**Figure 3:**
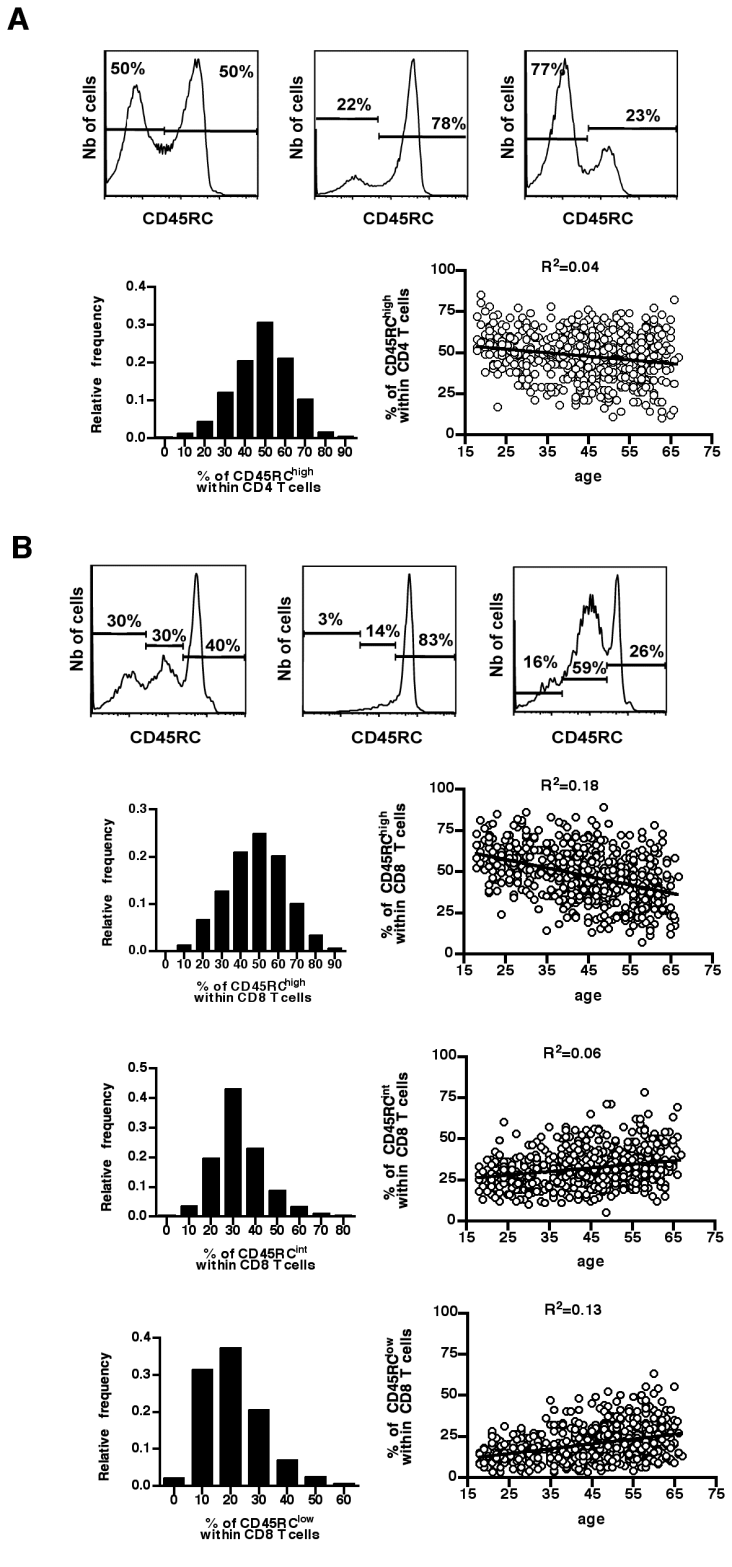
Distribution of CD4 and CD8 CD45RC T cell subsets in healthy individuals. Peripheral blood leukocytes from 608 healthy individuals were stained with mAbs against TCR, CD4, CD8 and CD45RC. The histograms represent CD45RC expression on CD4 T cells (**A**, top panels) and CD8 T cells (**B**, top panels) from three healthy individuals, to illustrate the inter-individual variability. The distribution of the proportion of CD45RC^high^ CD4 T cells (**A**) and of CD45RC^high^, CD45RC^int^ and CD45RC^low^ CD8 T cells (**B**) in the cohort of 608 healthy individuals is presented as histograms. The proportion of CD45RC subsets in CD4 (**A**) and CD8 (**B**) T cells is presented according to age and each dot represents a separate individual. The R^2^ values were calculated using linear regression.

In the rat model, it has been shown that the relative proportion of CD45RC subsets is highly variable between strains and that this variability is intrinsic to hematopoietic cells and is genetically controlled [12–14,26]. In humans, our previous study, using a limited numbers of Dutch healthy controls, showed an inter-individual variation of CD4 and CD8 CD45RC T cell subsets [11]. Here, we confirmed this observation using a large cohort of 608 healthy French individuals (259 women and 349 men, median age 45, range 18-67). Indeed, the proportion of the different subsets was highly variable within CD4 and CD8 T cells (CD4: Figure 3A; median and range for CD45RC^high^: 49% and 10-85%; CD8: Figure 3B; median and range for CD45RC^high^: 48% and 7-89%; CD45RC^int^: 31% and 5-78%; CD45RC^low^: 18% and 2-63%). The age of individuals had a minor impact on this variability since it accounted for only 4% of the variation within the CD4 compartment (Figure 3A) and, at best, for 18% of the variation within the CD8 compartment (Figure 3B).

#### The proportion of CD45RC subset predicts acute graft rejection

**Figure 4:**
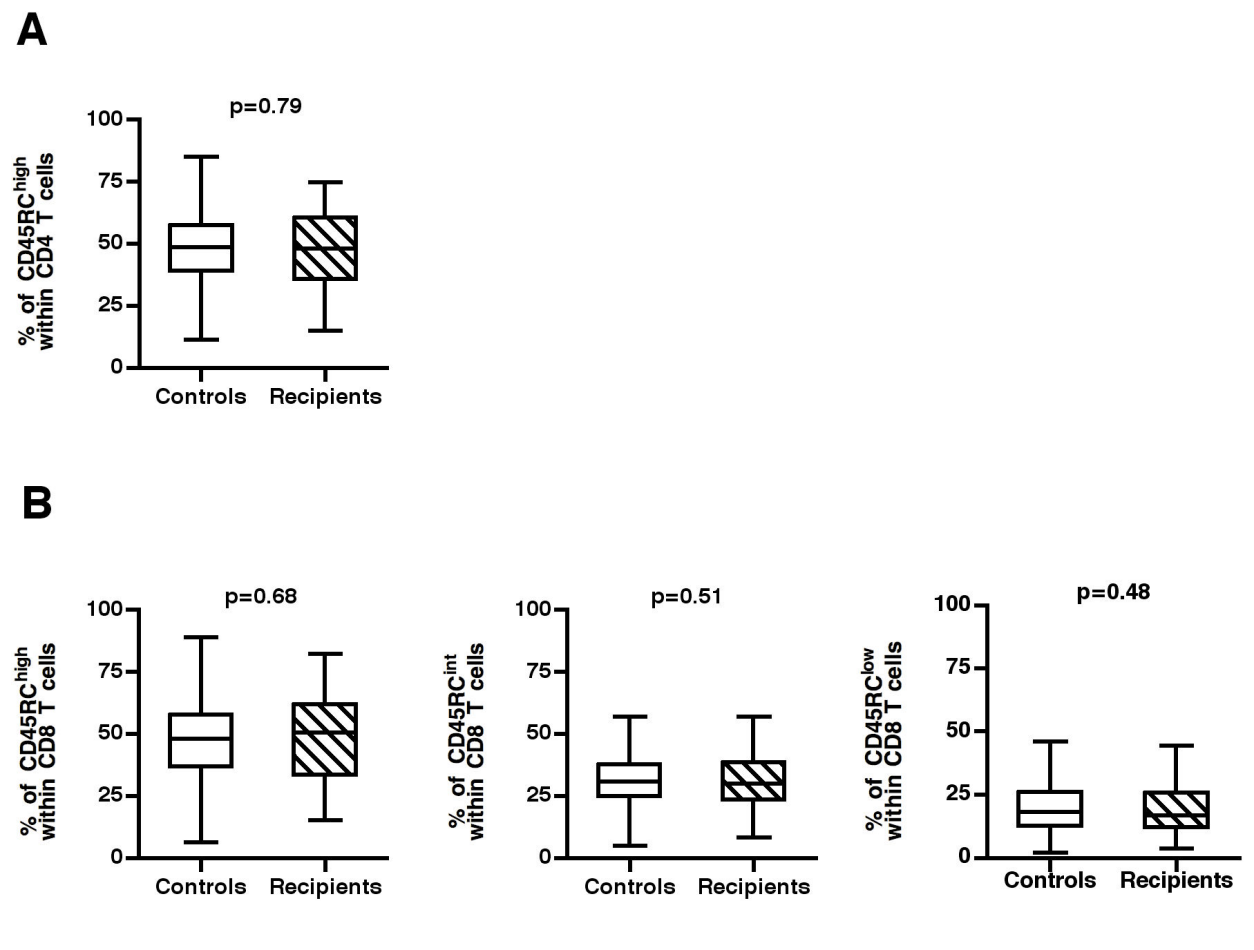
Distribution of CD45RC T cell subsets in the studied cohort compared to healthy individuals. Peripheral blood leucocytes from 89 patients were frozen in liquid nitrogen before renal transplantation. Thereafter, these cells were thawed and stained with mAbs against TCR, CD4, CD8 and CD45RC. (A, B) the proportion of CD45RC subsets within CD4 (A) and CD8 T cells (B) in the 89 recipient before the graft were compared to those of 608 healthy individuals. The p-values were calculated using the Mann-Whitney test.

The high variability of CD45RC T cell subsets between individuals, combined with the different *in vitro* alloreactive properties of CD45RC subsets, led us to hypothesize that the outcome of a graft could be different in transplanted patients with different pre-existing CD45RC profiles. To test this hypothesis, we analyzed whether the frequency of CD45RC T cell subsets before transplantation would predict acute graft rejection in a cohort of 89 patients who had undergone their first kidney transplantation. As shown in Figure 4, the general distribution of CD4 (Figure 4A) and CD8 (Figure 4B) CD45RC subsets in these patients was similar to that observed in the 608 healthy controls, indicating that kidney failure and dialysis had no impact on CD45RC expression by T cells.

**Figure 5:**
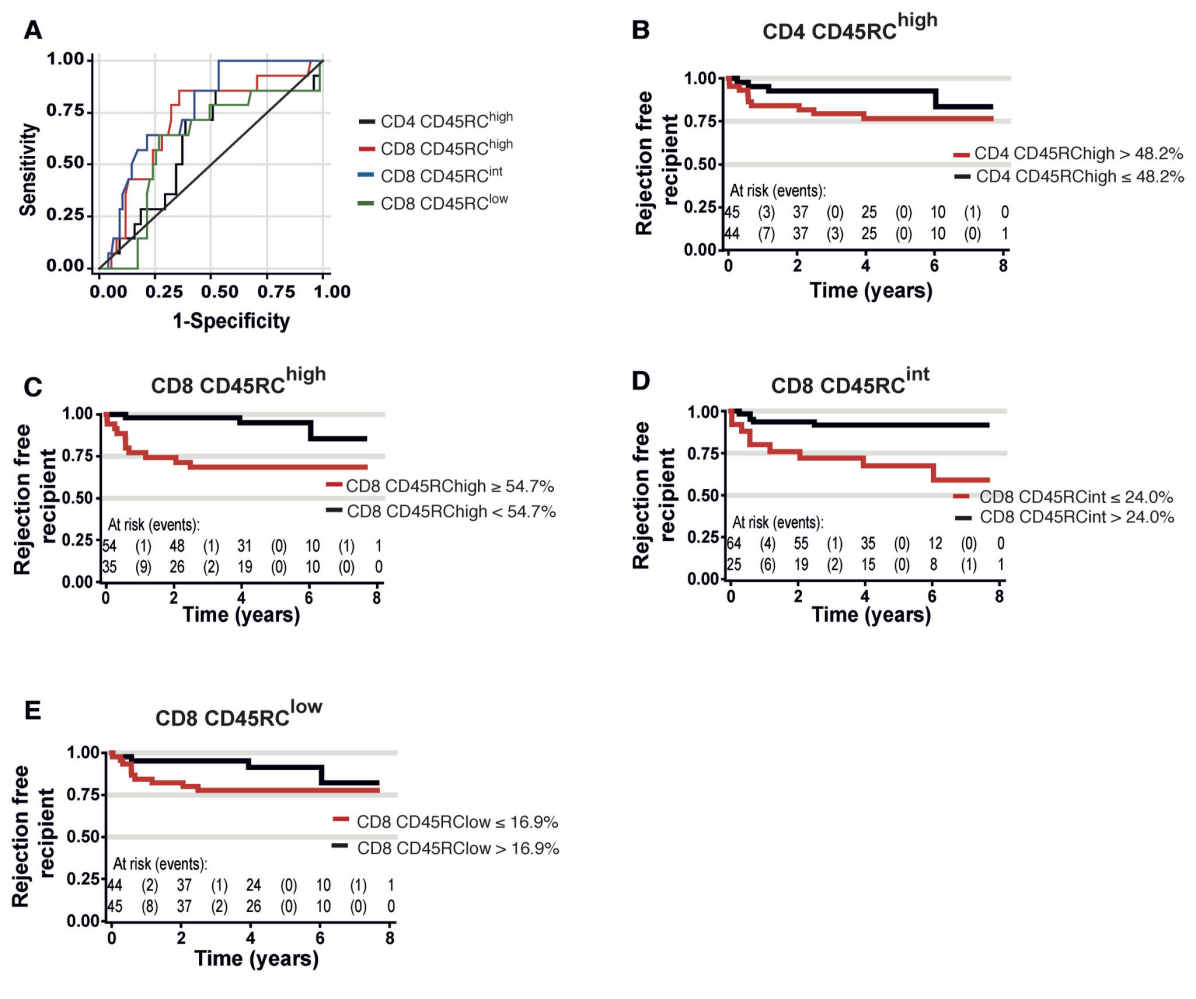
Proportion of CD45RC CD8 T cells before transplantation, a biomarker of acute kidney allograft rejection. (**A**) ROC curve analysis for CD45RC^high^ CD4 T cells (black), CD45RC^high^ CD8 T cells (red), CD45RC^int^ CD8 T cells (blue) and CD45RC^low^ CD8 T cells (green). To study CD45RC^high^ CD8 T cells (respectively CD45RC^high^ CD4 T cells), the number of true positives is the number of subjects who reject the transplant and who have a proportion of CD45RC^high^ CD8 T cells (respectively CD45RC^high^ CD4 T cells) higher or equal to the threshold value. To study CD45RC^int^ CD8 T cells (respectively CD45RC^low^ CD8 T cells), the number of true positives is the number of subjects who reject the transplant and who have a proportion of CD45RC^int^ CD8 T cells (respectively CD45RC^low^ CD8 T cells) lower or equal to the threshold value. (**B**, **C**, **D**, **E**) Kaplan-Meier curve estimates the survival without rejection based on CD45RC expression. The AUC for CD45RC^high^ CD4 T cell subset (0.60 CI95% [0.44–0.76]) and for CD45RC^low^ CD8 T cells (0.61 CI95% [0.44–0.77]) did not allow defining a threshold. The patients were therefore separated into two groups based on the median value for CD4 CD45RC^high^ and CD45RC^low^ CD8 T cells (**B**, **E**). However, the AUC for CD45RC^high^ and CD45RC^int^ CD8 T cell subset (0.71 CI95% [0.56–0.86] and 0.76 CI95% [0.65–0.88] respectively) permitted to define a threshold of 54.7% for CD45RC^high^ and 24.0% for CD45RC^int^. The patients were therefore separated into two groups based on these cut-off values obtained from ROC analyses for CD45RC^high^ (**C**) and CD45RC^int^ (**D**) within CD8 T cells.

The transplanted recipients (69 men, 20 women, median age 51, range 16-77 years) were followed for an average of 57.3 (range 0.03-98.9) months (Table 1). During this follow-up, 14 subjects exhibited acute graft rejection (overall acute rejection incidence of 15.73%) and the median occurrence was 6.9 months (range 0.3-72.5). In this cohort, acute graft rejection was not found to correlate with either the sex (HR 1.09 [0.30–3.90]; p=0.897), the initial anti-rejection therapy (type of induction: antithymocyte globulin versus basiliximab, HR 0.45 [0.15-1.37]; p=0.161), the type of treatment (monotherapy Tacrolimus versus mycophenolate mofetil/Tacrolimus, HR 0.54 [0.18-1.60]; p=0.268), the HLA mismatches (higher than 4, HR 1.79 [0.62-5.21]; p=0.284) or the cold ischemia time (higher than 18 hours, HR 2.08 [0.70-6.23]; p=0.188) (Table 2). To test whether the proportion of the different CD45RC subsets before transplantation could discriminate patients with or without rejection, we performed a Receiver Operating Characteristic (ROC)-curve analysis (Figure 5A). This analysis revealed that the proportions of CD45RC in CD8 T cell compartment were highly predictive of rejection, whilst those for CD4 T cells were not (Table 3 Figure 5B, C, D). Using the ROC curve, a threshold above 54.7% of CD8 CD45RC^high^ T cells before transplantation was identified as the best cut-off value (Figure 5A). Using this threshold, among the 35 patients with high numbers of CD45RC^high^ CD8 T cell, 11 rejected their graft, whereas in the 54 patients below the threshold, acute rejection only occurred in 3 of them (sensitivity=78.6%, specificity=68.0% and negative predictive value=94.4%) (Figure 5C). Therefore, patients with more than 54.7% of CD8 CD45RC^high^ T cells before transplantation had a six-fold higher risk to reject their kidney transplant than those below this threshold (HR 5.99; [1.67-21.51]; p=0.006) (Figure 5C Table 3).

**Table 1:** Characteristics of transplanted patients.

	**Total cohort (n=89)**	**Rejection (n=14)**	**No rejection (n=75)**	**p**
**Gender (Men/Women)**	69/20	11/3	58/17	1.0
**Age at transplantation in years**,				
median (IQR)	51 (37-59)	34 (25-48)	51 (41-60)	0.0076
**Cold ischaemia time (h)**				
median (IQR)	18 (15-21)	20.5 (12-23)	18 (15-21)	0.4194
**HLA mismatches,** median (IQR)				
**HLA-A**	1 (1-2)	1.5 (0-2)	1 (1-2)	0.6576
**HLA-B**	2 (1-2)	1.5 (1-2)	2 (1-2)	0.6120
**HLA-DR**	1 (1-2)	1 (1-2)	1 (1-2)	0.9700
**Total**	4 (3-5)	4 (3-5)	4 (3-5)	0.8502
**Panel reactive antibodies (%)**	0 (0-0)	0 (0-0)	0 (0-0)	0.3230
**Panel reactive antibodies N (%)**				
%Ab=0 / %Ab > 0	84/5	14/0	70/5	1.000
**Induction (No/basiliximab/ATG)**	8/26/55	0/6/8	8/20/47	0.318
**Monotherapy (No/Yes)**	32/57	6/8	26/49	0.558

Abbreviation: IQR, interquartile range; Ab, antibody; No induction, means that patients received only methylprednisolone; ATG, antithymocyte globulins Monotherapy means that patients received only Tacrolimus 6 months post-transplantation

**Table 2:** Crude hazard ratio (HR), 95% confidence interval (CI) and p-value (p) of recipient parameters on transplant rejection.

	**n**	**Number of rejection**	**HR (IC_95%_)**	**p**
**Gender**				
Women	20	3	1	
Men	69	11	1.09 (0.30–3.90)	0.897
**Age at transplantation in years**				
Age ≤ 51^a^	45	11	1	
Age > 51	44	3	0.28 (0.08–0.99)	0.049
**Cold ischaemia time (h)**				
≤18^a^	47	5	1	
>18	42	9	2.08 (0.70–6.23)	0.188
**HLA mismatches**				
Total ≤4^a^	60	8	1	
Total >4	29	6	1.79 (0.62–5.21)	0.284
**Induction**				
No	8	0		
basiliximab	26	6	1^b^	
ATG	55	8	0.45 (0.15–1.37)	0.161
**Monotherapy**				
No	32	6	1	
Yes	57	8	0.54 (0.18–1.60)	0.268

a Subgroups of patients were defined using the median values of the cohort.

b patients with no induction are excluded from this bivariate analysis

Abbreviation: HR, Hazard Ratio; CI, confidence interval; No induction means that patients received only methylprednisolone; ATG, antithymocyte globulins Monotherapy means that patients received only Tacrolimus 6 months post-transplantation

**Table 3:** Crude hazard ratio (HR), 95% confidence interval (CI) and p-value (p) of different human T-cell subsets according on transplant rejection.

	**n**	**Number of rejection**	**HR (CI_95%_)**	**p**
**CD4 T cells**				
% CD45RC^high^ ≤ 48.2	45	4	1	
> 48.2	44	10	2.52 (0.79–8.04)	0.118
%RA+RO-CCR7+ ≤ 36.5	45	6	1	
> 36.5	44	8	1.29 (0.45–3.73)	0.636
%RA-RO+CCR7+ ≤ 29.8	45	9	1	
> 29.8	44	5	0.58 (0.19–1.72)	0.326
%RA-RO+CCR7- ≤ 14.3	45	9	1	
> 14.3	44	5	0.60 (0.20–1.78)	0.354
**CD8 T cells**				
% CD45RC^high^ < 54.7^a^	54	3	**1**	
≥ 54.7	35	11	**5.99 (1.67–21.51)**	**0.006**
% CD45RCint ≥ 24.0^a^	64	5	**1**	
< 24.0	25	9	**4.75 (1.59–14.23)**	**0.005**
% CD45RC^low^ ≤ 16.9	45	10	1	
> 16.9	44	4	0.41 (0.13–1.31)	0.134
%RA+RO-CCR7+ < 36.1^a^	50	3	**1**	
≥ 36.1	39	11	**4.71 (1.31–16.92)**	**0.017**
%RA-RO+CCR7+ ≤ 6	46	7	1	
>6	43	7	1.15 (0.40–3.28)	0.791
%RA-RO+CCR7- ≤ 10.6	45	7	1	
> 10.6	44	7	1.10 (0.38–3.13)	0.861

a For these variables, the subsets were dichotomized using the threshold obtained by the ROC curve rather than the median.

Abbreviation: HR, Hazard Ratio; CI, confidence interval

Conversely, we found that low proportions of CD8 CD45RC^int^ also showed predictive value for rejection: patients with less than 24.0% of CD8 CD45RC^int^ cells had almost a five-fold higher risk to suffer from acute rejection than those with more value above that threshold (HR 4.75 [1.59-14.2]; p=0.005) (Figure 5D Table 3). The sensitivity, specificity and negative predictive values were respectively of 64.3%, 78.7% and 92.2% (Figure 5A). In contrast, CD8 CD45RC^low^ cells were not significantly associated to acute rejection (HR 0.41 [0.13-1.31]) (Figure 5E Table 3). Together, our results demonstrate that the level of CD45RC expression on CD8 T cells could be used to identify patients with higher risk of acute rejection of human kidney allografts.

Peripheral T cells can be broadly divided into naive (CD45RA+ RO-CCR7+) and antigen-experienced T cells, with the latter including effector (CD45RA-RO+CCR7-) and central memory (CD45RA-RO+CCR7+) T cells. We previously showed that the CD8 CD45RC^low^ subset contains mainly central (CD45RA-RO+CCR7+) and effector memory (CD45RA-RO+CCR7-) T cells while the CD8 CD45RC^high^ subset contains mainly naive cells (CD45RA+ RO-CCR7+) [11]. In the present study, we tested whether the proportion of these functionally distinct CD8 subsets before transplantation could also discriminate patients with or without rejection. We found that the proportion of naïve CD8 T cells (CD45RA+ RO-CCR7+) were predictive of rejection. Using the ROC curve, a threshold above 36.1% of CD8 CD45RA+ RO-CCR7+ T cells before transplantation was identified as the best cut-off value. Using this threshold, among the 39 patients with high numbers of CD8 CD45RA+ RO-CCR7+ T cells, 11 rejected their graft, whereas in the 50 patients below the threshold, acute rejection only occurred in 3 of them (sensitivity=78.6%, specificity=62.7% and negative predictive value=94%). Therefore, patients with more than 36.1% of CD8 CD45RA+ RO-CCR7+ T cells before transplantation had a five-fold higher risk to reject their kidney transplant than those below this threshold (HR 4.71; [1.31-16.92]; p=0.017) (Table 3). In contrast, effector memory CD8 T cells (CD45RA-RO+CCR7-) and central memory CD8 T cells (CD45RA-RO+CCR7 were not significantly associated to acute rejection (HR 1.15 [0.40-3.28]-p=0.791 and HR 1.10 [0.38-3.13]-p=0.861 respectively) (Table 3). The analysis of Foxp3 expression by CD8 T cells show that the majority of Foxp3+ CD8 T cells are contained in the CD45RC^low^ subset [11] and this marker is not predictive for graft rejection (data not shown). Together, these data show that, among all markers tested, the CD45RC is clearly the best biomarkers for predicting kidney allograft rejection.

## Discussion

In the present study, we show that human CD45RC CD4 and CD8 T cell subsets display distinct alloreactive properties *in vitro*. In response to direct allogeneic presentation, they exhibit differences in their proliferative ability and in their cytokine profiles. Moreover, the proportions of these T cell sub-populations, analyzed in a large French cohort of 608 healthy individuals, are highly variable between individuals independently of age. Importantly, we show in a cohort of recipients of kidney transplants that individuals harboring a high frequency of CD45RC^high^ CD8 T cells before transplantation have a six times higher risk to develop acute kidney graft rejection. These data imply that pre-transplant frequency of CD45RC^high^ CD8 T cells in a given host before transplantation may be used to evaluate the risk of acute rejection and to adapt the immunosuppression treatment accordingly.

The cytokinic profile of immune cells, which is tightly controlled in a complex manner, plays a central role in orchestrating the outcome of alloreactive immune responses [27–33]. The difference in cytokine profiles of CD45RC T cell subsets in response to alloantigen stimulations might explain how different proportions of these subsets could influence a patient’s response to an allograft. The type 1 cytokines, in particular IFN-γ, which are key mediators of graft rejection, are mainly produced by the CD45RC^high^ CD8 T cell subset [27–29]. In contrast, the CD8 CD45RC^low^ T cells have the capacity to recognize alloantigens, but produce anti-inflammatory cytokines, in particular IL-10, a key cytokine that controls graft rejection and graft versus host disease (GvHD) [29–32]. Interestingly, this subset is also characterized by a high production of IL-17. In the past decade, many studies in animal models and clinical transplantation have demonstrated that IL-17 is involved in allograft rejection [33]. However, the involvement of IL-17 in graft rejection is still a matter of debate [34,35], and there is evidence showing that cells producing IL-17 could play a regulatory role in alloreactive and inflammatory responses [35–37]. In these models, IL-17 seems to control the type 1 and type 2 responses responsible for these pathologies. This protective role of IL-17 could also be explained by the fact that regulatory T cells also produce IL-17 [38,39]. Together, these data are consistent with the hypothesis that CD45RC^low^ CD8 T cells have retained their ability to recognize alloantigen but are but are bereft of effector activity involved in the graft rejection. In contrast to the CD8 T cell subsets, differential CD45RC expression among CD4 T cells did not predict graft rejection. This could be explained by the capacity of these subsets to produce both inflammatory and anti-inflammatory cytokines.

Regulatory T cells are characterized by the expression of the transcription factor, Foxp3, and play a central role in preventing pathological immune responses including autoimmunity, allergy, and transplantation [40–42]. The difference between CD45RC T cell subsets in their ability to predict graft rejection could also be explained by their difference in regulatory T cell composition. Indeed, both in rodent and in human, the majority of cells expressing Foxp3 are contained within the CD45RC^low^ population [11,20]. In addition, in rats, the CD45RC^low^ CD8 T cell subset, but not the CD4 CD45RC^low^ T cells, inhibit GvHD [18,20] and heart allograft rejection [21].

Peripheral T cells can be broadly divided into those that have never been activated by antigen (naive T cells) and antigen-experienced T cells, which include effector, central memory, and effector memory cells. The best-characterized phenotypic criteria to discriminate these distinct stages of post-thymic human T-cell differentiation are the expression level of CD45RA, CD45RO and CCR7 [43,44] By using these markers, we previously showed that the CD45RC^low^ subset contains mainly central (CD45RA-RO+CCR7+) and effector memory (CD45RA-RO+CCR7-) cells while the the CD45RC^high^ subset contains mainly naive cells (CD45RA+ RO-CCR7+) [11]. In human and non-human primates, it has been shown that memory T cells responding to environmental antigens can exhibit cross-reactivity with donor alloantigens through various mechanisms collectively known as heterologous immunity [45,46]. It has been suggested that individuals with a higher precursor frequency of donor-reactive memory T cells are at increased risk of developing acute allograft rejection after transplantation [47]. In Table 3, we show that the proportion of naive cells (CD45RA+ CD45RO-CCR7 +) among CD8 T cells was the only sub-population that correlates with the probability of rejection of a transplant (HR 4.71 [1.31–16.92]; p=0.017). This is in agreement with studies in rodents showing that T cells with naive phenotype induce a deleterious alloreactive immune response that induce GvHD, in contrast to total memory T cells or purified central memory T cells, which are unable to promote this disease [18,48–51]. The incapacity of memory T cells to induce a pathogenic alloreactive response has been attributed either to their restricted repertoire or to their difference in migration capacity and tissue localization [51]. Of note, the CD45RC is clearly the best of the four biomarkers for predicting kidney allograft rejection (Table 3). The age was also found to have a predictive value but not as strong as the level of CD45RC expression on CD8 T cells. It seems likely, however, that the combination of these various factors could increase the predictive value, but this type of analysis will require much larger cohorts that the one we had at our disposal for the current study.

Although many advances have been made in the field of transplantation, anti-rejection therapy is far from ideal. The current standard of care for transplant recipients is the immunosuppressive treatments for life that are associated with heavy side effects such as nephrotoxicity, hypertension, cardiovascular disease, opportunistic infections and malignancies. These side effects could be reduced if biomarkers were available to allow individual tailoring of potentially toxic immunosuppressive therapy. Recent studies have identified biomarker signature associated with allograft tolerance, and allowed certain patients to stop the immunosuppressive treatment and still have a stable kidney transplant [52,53]. To date, however, there are still no adequate markers that can be used before transplantation to predict the probability of graft rejection in order to adapt the immunosuppressive treatments as early as possible to avoid the serious side effect of these drugs. The present study shows that the level of CD45RC expression on recipient CD8 T cells before transplantation represents a very promising candidate for such a biological marker. This marker has several advantages since a) it requires a minimally invasive procedure (blood testing), b) it can be easily implemented in routine immunology laboratories, c) it would be evaluated before the transplantation and could have an impact on the choice of the immunosuppressive regimen employed immediately after grafting, as well as for tapering off the immunosuppressive treatment. Thus, knowing the percentage of CD45RC subsets within CD8 T cells in potential transplant recipients could help the transplant centers to improve the treatment, and ultimately the quality of life of the transplanted patients.
